# Repurposing of known anti-virals as potential inhibitors for SARS-CoV-2 main protease using molecular docking analysis

**DOI:** 10.6026/97320630016301

**Published:** 2020-04-30

**Authors:** Mohammed Hakmi, El Mehdi Bouricha, Ilham Kandoussi, Jaouad El Harti, Azeddine Ibrahimi

**Affiliations:** 1 Medical Biotechnology Laboratory (MedBiotech), Rabat Medical and Pharmacy School, Mohammed Vth University in Rabat, Morocco; 2 Therapeutic Chemistry Laboratory, Medical Biotechnology Laboratory (MedBiotech), Rabat Medical and Pharmacy School, Mohammed Vth University in Rabat, Morocco

**Keywords:** COVID-19, SARS-Cov-2, protease, inhibitors, virtual screening

## Abstract

The new SARS-CoV-2 coronavirus is the causative agent of the COVID-19 pandemic outbreak that affected more than 190 countries worldwide with more than 292,000 confirmed cases and over 12,700 deaths. There is at the moment no vaccine or effective treatment for this disease which constitutes a serious global health problem. It is of interest to use a structure based virtual screening approach for the identification of potential inhibitors of the main protease of SARS-CoV-2 (M^pro^) from antiviral drugs used to treat other viral disease such as human immunodeficiency virus (HIV) and hepatitis C virus (HCV) infections. The crystallographic structure with PDB ID: 6LU7 of M^pro^ in complex with the inhibitor N3 was used as a model in the virtual screening of 33 protease inhibitors collected from the ChEMBL chemical database. Molecular docking analysis was performed using the standard AutoDock vina protocol followed by ranking and selection of compounds based on their binding affinity. We report 10 candidates with optimal binding features to the active site of the protease for further consideration as potential drugs to treat patients infected with the emerging COVID-19 disease.

## Background:

The coronavirus disease 2019 (COVID-19) began in late 2019 in Wuhan in China's central province of Hubei [1] and the infection is caused by a novel coronavirus (SARS-CoV-2) that has been isolated from patients presenting a mysterious atypical severe pneumonia [2]. The new virus is believed to be very contagious and has already spread rapidly around the world [3]. As of March 23, 2020, the COVID-19 pandemic has affected over 190 countries and territories, with more than 464,142 laboratory-confirmed cases and 21,100 deaths [4]. At the time this paper was written, there have been no specific or effective drugs for the treatment or prevention of COVID-19. Therefore, proposals for the development of new drugs are of fundamental importance in this global health emergency. A wide range of compounds, vaccines and biologics are being investigated by scientists all around the world as potential therapies for COVID-19 [5,6]. One of the common drug targets encoded by the viral genome of SARS-CoV-2 is its main protease (M^pro^), which plays a critical role in the growth and spread of the virus. Protease activity can be neutralized by antiviral drugs, known as protease inhibitors, that selectively bind to the catalytic site of the enzyme and prevent the production of infectious virions [7]. High-resolution crystal structure of the SARS-CoV-2 M^pro^ in complex with a covalently bound inhibitor has been recently been published with PDB ID: 6LU7 [8] and made available to the scientific community, which can be seen as a real opportunity for modern drug discovery initiatives for COVID-19. We have thus implemented a structure-based virtual screening (SBVS) approach to repurpose available antiviral drugs as potential treatments for COVID-19. Drug repositioning is a proven, cost-effective and time saving solution for finding new indications for already established drugs. Our strategy has focused on the identification of small molecules with potential activity against SARS-CoV-2 M^pro^ from a subset of protease inhibitors intended for the treatment or management of other viral infections such as those caused by the hepatitis C virus (HCV) or the human immunodeficiency virus (HIV).

## Materials and Methods:

### Collection of protease inhibitor drugs:

A search for "Protease Inhibitors" was conducted in the ChEMBL 26 database [9, 10] to list all protease inhibitor drugs available at the time of this study. During the analysis, we selected the "compounds" tab and applied the "small molecules" filter to keep only small molecule drugs. The product ingredients were rejected and a total of 33 2D structures of small molecule inhibitors were downloaded in a GZipped SD file format (SDF).

### Preparation of drugs structures:

The archive downloaded in the previous step was extracted using PeaZip (release 7.1.0-win64 build) and the imperfections in the initial SDF were corrected by opening it in OpenBabel [11] (version 3.0.0 GUI ) and saving it as a new SDF. The latter was then imported into Vconf (Vconf interface UI 2.0 for windows) [12] for preparation. The 2D to 3D preparation ("prep") mode was used to convert the initial 2D conformations of each molecule into high-quality 3D structures with distinct conformations. This mode is well suited for the preparation of molecules for the ulterior calculations that imply varying bond torsions, such as docking.The resulting 3D SDF from Vconf was converted into separate PDBQT files using OpenBabel.

### Preparation of receptor structure:

Three-dimensional structure file (PDB code: 6LU7, resolution 2.16 Å) of the SARS-CoV-2 Mpro was downloaded from the RCSB PDB database [13] and prepared in AutoDockTools [14] (ADT version 1.5.7rc1) by removal of water and solvent molecules, removal of the bound ligand, addition of polar hydrogens and partial charge assignment. The prepared structure was saved in AutoDock PDBQT format. The co-crystallized ligand (http://www.rcsb.org/bird/PRD_002214) of the 6LU7 structure was extracted, prepared and saved in PDBQT format using ADT to be included as a reference in the virtual screening.

### Virtual screening procedure:

The docking simulations were performed using AutoDock vina 1.1.2 [15]. The center (-10,782, 15,787, 71,277) of the search space has been determined on the basis of the co-crystallized bound peptidomimetic ligand, and its size has been set to 20x20x20 Angstroms to cover the active site of the protease. The number of solutions has been fixed to 10 while the remaining of AutoDock vina parameters have been kept at their default values. The virtual screening experiment was conducted using an in-house developed python script.

### Post docking analysis:

The results of the virtual screening experiment were ranked according to the binding energy of their best scoring conformation. The top ranked 10 candidates were selected for further analysis. Visual inspection of docking poses and the analysis of protein-ligand interactions were performed in Biovia Discovery Studio Visualizer version 20.1.0.19295 (Dassault systemes Biovia corp). Visualization images were rendered by PyMOL 2.3 (Schrodinger L.L.C).

## Results and discussion:

**Figure 1:**
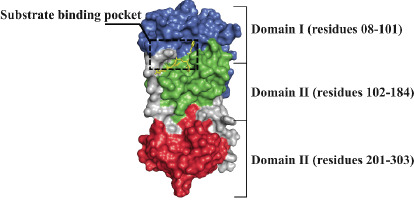
Surface representation of SARS-CoV-2 M^pro^ protomer showing its domain organization (PDB code 6LU7). The substrate binding site is located in the pocket between domain I (blue) and II (green). Domain III is colored in red and the co-crystallized bound inhibitor N3 is represented by yellow sticks.

With the publication of the SARS-CoV-2 viral genome in January 2020 [16], key components of the virus have been identified to drug designers to look for potential drugs to stop the spread of the virus. Of these components, M^pro^ is most often targeted as it plays a major role in controlling the virus' self-replicating machinery. SARS-CoV-2 M^pro^ is a cysteine protease homodimer formed by two identical protomers. As shown in Figure 1, each protomer consists of three domains and a substrate-binding site containing the catalytic cysteine-histidine dyad. The substrate binding pocket is located between domains I and II of each protomer [8].

In this work, we hypothesized that protease inhibitors designed to deal with other viruses such as HIV and HCV may also have some activity on M^pro^. To verify this, we performed a virtual screening of 33 protease inhibitors that we collected from the ChEMBL chemical database. In addition to the drugs already approved, we decided to include experimental compounds, which can serve as prototypes for the development of new compounds, which would be more effective in the treatment of COVID-19. The top-ranked 10 candidates from our virtual screening experiments on the M^pro^ of SARS-CoV-2 are presented in Table 1.

**Figure 2:**
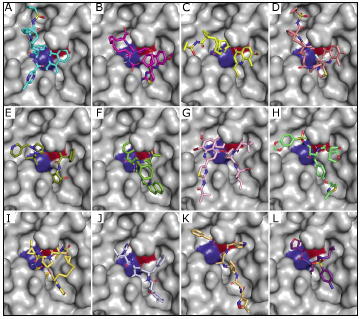
Binding modes of the ten selected candidates in the substrate-binding site of SARS-CoV-2 M^pro^. (A) Paritaprevir; (B) Ciluprevir; (C) Simeprevir; (D) Deldeprevir; (E) Indinavir; (F) Saquinavir; (G) Faldaprevir; (H) Brecanavir; (I) Grazoprevir; (J) Lopinavir; (K) Inhibitor N3; (L) Darunavir. The catalytic dyad Cys145-His41 is colored in red and blue respectively.

**Figure 3:**
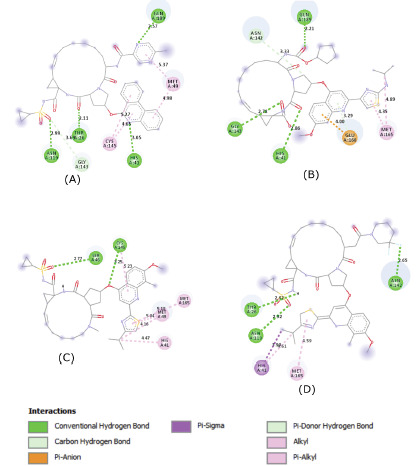
Molecular contacts between the selected candidates and the residues of the substrate-binding pocket of SARS-CoV-2 M^pro^.(A), Paritaprevir.(B),Ciluprevir.(C), Simeprevir. (D), Deldeprevir. The four compounds established multiple hydrogen bonding and hydrophobic interaction with the residues of the substrate-binding pocket of M^pro^. Interactions with the catalytic His41 were observed in the four compounds. Paritaprevir and Simeprevir interacted with the catalytic Cys145 as well throught a hydrophobic contact and a hydrogen bond respectively.

**Figure 4:**
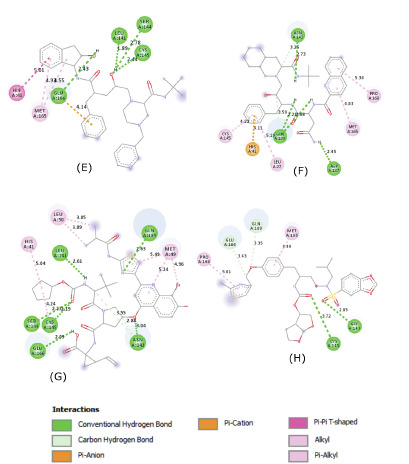
Molecular contacts between the selected candidates and the residues of the substrate binding pocket of SARS-CoV-2 M^pro^.(E),Indinavir.(F),Saquinavir.(G),Faldaprevir. (H), Brecanavir. The four compounds were stabilized inside the substrate-binding pocket of M^pro^ by a network of hydrogen bonds and hydrophobic contacts. Interactions with the catalytic dyad Cis-145-His41 were observed in Indinavir, Saquinavir and Faldaprevir; however, Brecanavir interacted only with the catalytic Cys145.

**Figure 5:**
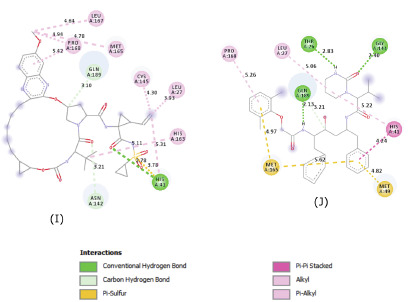
Molecular contacts between the selected candidates and the residues of the substrate binding pocket of SARS-CoV-2 M^pro^.(I),Grazoprevir.(J),Lopinavir. More hydrophobic interactions than hydrogen bonds were observed in Grazoprevir, which interacted with both catalytic residues Cys145 (1 hydrophobic contact) and His41 (1 hydrogen bond and 1 Pi sulfur bond). Lopinavir interacted only with His-41 through a Pi-Pi stacked interaction and a hydrophobic contact.

The co-crystallized peptidomimetic ligand known as inhibitor N3 in the 6LU7 structure as well as Darunavir, a second-generation anti-HIV-1 protease inhibitor [17] that is currently under clinical trials as a potential treatment for SARS-CoV-2 [18], were taken as references in the virtual screening experiment conducted on 6LU7 structure of SARS-CoV-2 M^pro^. Inhibitor N3 and Darunavir had respectively a binding energy of -7.0 kcal/mol and -7.4 kcal/mol. The top-ranked 10 candidates all showed better binding affinities to the active site of M^pro^ as compared to inhibitor N3 and Darunavir. The binding energy of our selected candidates ranged from -8.1 kcal/mol to -9.5 kcal/mol with the lowest affinity for Lopinavir and the highest for Paritaprevir. It should be noted that lopinavir, presented here as one of the least binding candidates, was used in combination with ritonavir to treat patients with severe Covid-19 in a randomized controlled trial but the results were found to be unsatisfactory and without any therapeutic efficacy [19]. Despite this, visual inspection of the predicted binding modes showed that the selected candidates were reasonably anchored within the substrate binding pocket of Mpro and eventually occupied the pocket in the same manner as the co-crystallized inhibitor N3 (Figure 2). Detailed examination of atomic interactions at protein-ligand interfaces indicated the formation of multiple hydrogen bonds, hydrophobic interactions and Pi interactions that may help to stabilize the ligands inside the substrate-binding site (Figure 3, Figure 4 and Figure 5).

Remarkably, all of the proposed candidates interacted with one or both catalytic residues (Cys145 and His41) in the substrate binding site of SARS-Cov-2 M^pro^ [8]. This site is homologous in all coronavirus proteases and therefore these compounds may have a potential inhibitory activity on other corona viruses as well. As these results appear very promising, further bioassays and clinical trials are needed to confirm the inhibitory activity of these compounds against SARS-Cov-2 main protease.

## Conclusions:

In this paper, we report 10 candidates from known anti-viral drugs with optimal binding features to the active site of the protease for further consideration to fight the COVID-19 pandemic and the care for the infected persons. With the increasing number of sudden outbreaks of infectious diseases that threaten our global health, drug repurposing can be a cost effective and time-efficient strategy for the treatment and control of the emerging pathogens. The availability of pharmacokinetic evidence allows approved drugs to move quickly through the final stages of clinical trials, enabling a rapid response that can save hundreds or even thousands of lives.

## Figures and Tables

**Table 1:** The top 10 candidates ranked by binding affinity in the virtual screening of 33 protease inhibitor drugs against the SARS-CoV-2 M^pro^ 6LU7 structure.

Compound	ChEMBL ID	Phase	Primary indication	Binding energy (kcal/mol)
Paritaprevir	CHEMBL3391662	Approved	Treatment of HCV	-9,5
Ciluprevir	CHEMBL297884	Experimental	Treatment of HCV	-9,1
Simeprevir	CHEMBL501849	Approved	Treatment of HCV	-9,0
Deldeprevir	CHEMBL3040582	Experimental	Treatment of HCV	-8,6
Indinavir	CHEMBL115	Approved	Treatment of HIV	-8,5
Saquinavir	CHEMBL114	Approved	Treatment of HIV	-8,5
Faldaprevir	CHEMBL1241348	Experimental	Treatment of HCV	-8,4
Brecanavir	CHEMBL206031	Experimental	Treatment of HIV	-8,1
Grazoprevir	CHEMBL2063090	Approved	Treatment of HCV	-8,1
Lopinavir	CHEMBL729	Approved	Treatment of HIV	-8,1
